# The brain-inspired decoder for natural visual image reconstruction

**DOI:** 10.3389/fnins.2023.1130606

**Published:** 2023-05-02

**Authors:** Wenyi Li, Shengjie Zheng, Yufan Liao, Rongqi Hong, Chenggang He, Weiliang Chen, Chunshan Deng, Xiaojian Li

**Affiliations:** ^1^Brain Cognition and Brain Disease Institute (BCBDI), Shenzhen-Hong Kong Institute of Brain Science-Shenzhen Fundamental Research Institutions, CAS Key Laboratory of Brain Connectome and Manipulation, Shenzhen Institute of Advanced Technology, Chinese Academy of Sciences, Shenzhen, China; ^2^University of Chinese Academy of Sciences, Beijing, China; ^3^Clinical Medicine Institute, Chengdu University of Traditional Chinese Medicine, Chengdu, China; ^4^Illinois Institute of Technology, Chicago, IL, United States

**Keywords:** neural decoder, image reconstitution, brain-inspired ANNs, loss functions, autoencoder

## Abstract

The visual system provides a valuable model for studying the working mechanisms of sensory processing and high-level consciousness. A significant challenge in this field is the reconstruction of images from decoded neural activity, which could not only test the accuracy of our understanding of the visual system but also provide a practical tool for solving real-world problems. Although recent advances in deep learning have improved the decoding of neural spike trains, little attention has been paid to the underlying mechanisms of the visual system. To address this issue, we propose a deep learning neural network architecture that incorporates the biological properties of the visual system, such as receptive fields, to reconstruct visual images from spike trains. Our model outperforms current models and has been evaluated on different datasets from both retinal ganglion cells (RGCs) and the primary visual cortex (V1) neural spikes. Our model demonstrated the great potential of brain-inspired algorithms to solve a challenge that our brain solves.

## 1. Introduction

Brain–computer interface clinical studies have made remarkable achievements in recent decades, and brain activity decoding contributes significantly to the successes (Nishimoto et al., 2011; Gaziv et al., 2022). Brain activity decoding (or “brain reading”) is a vital theory to understand the brain's working mechanism, and the BCI application in practice mentioned earlier (Kay et al., 2008; Miyawaki et al., 2008; Rubin et al., 2017). Spike trains are the gold standard of neural activity. They are generated by single neurons that receive and respond to input stimuli by changing their membrane potential to generate a sequence of related events. Spike trains probably contain a basic unit of neural computation for a different task and corresponding features of neural computation at different neural network levels (Zador, 1997; Simoncelli and Olshausen, 2001; Wu et al., 2006). Spike trains of the neural population were widely used for motor intention decoding in BCI research (Andalib et al., 2019), both in animals and patients, demonstrating its potential in another form of decoding, such as images (Hayashi and Kawata, 2018; Ran et al., 2021; Li et al., 2022). The organization of our visual system is hierarchical, which means that the receptive fields of neurons at one level are constructed by combining inputs from neurons at a lower level (Grill-Spector and Malach, 2004). Thus, after several processing stages, small receptive fields tuned to simple stimuli combine to form larger receptive fields tuned to more complex stimuli (Serre, 2014). Research has shown that the distribution of the receptive field is asymptotically Gaussian (Luo et al., 2016). More specifically, neural spikes in the early stage, like retinal ganglion and primary visual cortex (V1) neurons, are tuned mostly on physical features of visual stimuli, such as luminance, contrast, orientation, and spatial frequency (Dai and Wang, 2012); while in the late stage, such as in inferior temporal cortex neurons, preferred mostly on psychological features of visual stimuli, such as face identity, and emotion (Baron, 1981; Schupp et al., 2003). Although there are many methods to decode the brain signal, it is still difficult to decode the spike signal in the V1 brain region. Moreover, current methods lack a biological basis. Therefore, we combined receptive field properties into an end-to-end neural network and trained the neural network using spike trains from both RGC and V1 neurons. The results showed that our model with biological theory outperforms current other models, demonstrating the remarkable potential of a brain-inspired algorithm.

## 2. Materials and methods

### 2.1. Datasets

#### 2.1.1. Datasets from monkey V1

**Figure 1:**
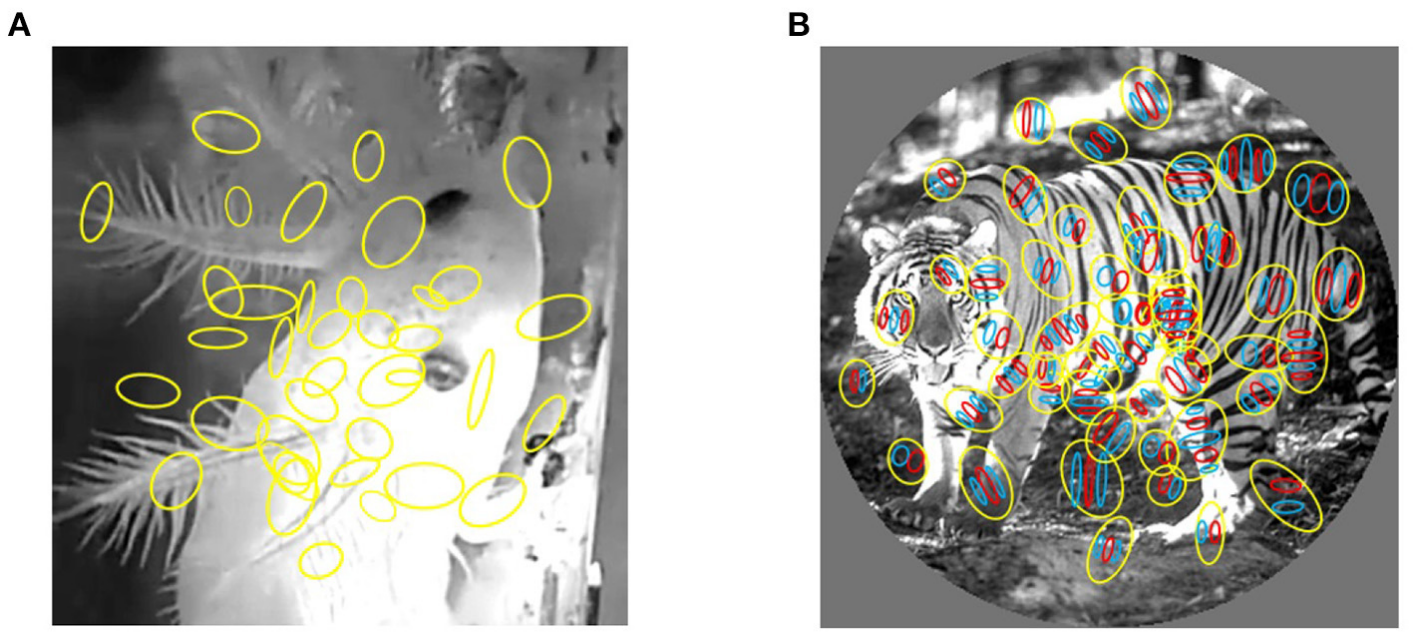
Mapping of receptive fields to video frames. **(A)** Receptive fields of salamander RGCs. **(B)** The receptive fields of macaque primary visual cortex neurons. Each yellow colored circle is an outline of receptive field. The red and blue region represent excitatory and inhibitory subregions, respectively.

We tested our model on two natural neural spike train datasets evoked by natural images. The first datasets are macaque V1 datasets which consist of multi-electrode recordings from V1 in anesthetized macaque using multi-electrode array (namely, Utah array, USA). At the same time, natural images and gratings were presented on the screen in front of the monkeys. Natural images were shown in two sizes, 3–6.7 degrees and windowed to 1 degree, to quantify surround modulation. The receptive field was measured using small gratings presented at various positions. The receptive field center of each neuron was defined as the location of the peak of a 2D Gaussian fit to the spatial activity map. The data were collected in the Laboratory of Adam Kohn at the Albert Einstein College of Medicine and downloaded from the CRCNS website. Experimental procedures and stimuli are fully described in the associated article (Coen-Cagli et al., 2015). The receptive field position relative to the image center is included in the data files and the ON/OFF receptive fields of each cell were computed by correlating their responses with the locations of bright and dark parts in the stimuli at different time delays (Figure 1) (Tring et al., 2022; Vafaei et al., 2022).

#### 2.1.2. Datasets from salamander retina

The second dataset consists of temporal firing recordings from 49 retinal ganglion cells (RGCs) of salamanders, while videos were projected onto the retina through a telecentric lens. The video stimuli comprised 1,800 frames and were made up of a 60-s-long natural movie clips presented at a frame rate of 30 Hz. The pixel size was resized to 64*64 pixels. There were 1800 video frames used as stimuli, and the dataset included the spike trains of 49 RGCs as responses to these video frames. The training set contained 1,440 (1800*0.8) video frames of 64*64 pixels, and the test set contained 360 (1800*0.2) video frames. These datasets and descriptions can be found in this article (Onken et al., 2016). Our decoder was used to reconstruct video frames from the spike trains of a population of RGCs of salamanders.

### 2.2. Data process

For the macaque V1 datasets, seven session data from multiple experiments were used. We used 80% data (1,249) as the training set and the remaining 20% data as the test set. We intercepted a small part of the data to ensure that each batch input dimension is equal. The data of 100 neurons in 105 milliseconds (ms) were retained every session and time 0 is the onset of the stimulus. The reconstructed grating images are modified to 80*80 pixels grayscale, and the natural images are reduced to 32*32 pixels grayscale. Moreover, as to the salamander's retina datasets, we converted the timestamp to spike trains on a 10 ms scale. We randomly selected some scenes and disrupted the order to wash out the temporal correlation within the video.

### 2.3. Model

**Figure 2:**
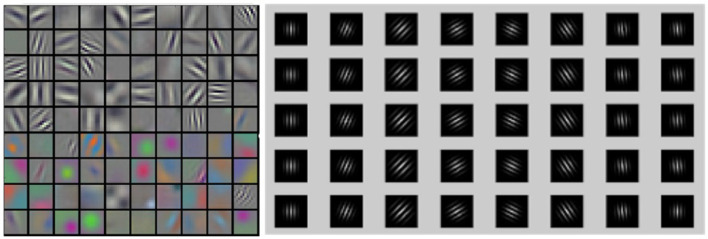
Comparison between CNN filters and Gabor filters. Latent features illustration of CNN filters **(left)** and Gabor filters **(right)**.

In this study, the structure of the auto-encoder is used (Hinton and Salakhutdinov, 2006). The network structure uses LeakyReLU as the activation function, with a learning rate of 0.02 that decays by a factor of 0.9 every 100 epochs. The network is optimized using an Adam optimizer, and a dropout of 0.5 is applied. The first layer of the network receives spikes from all neurons as input, and the second layer is an output layer that matches the image size. The encoder consists of four layers, with batch sizes of 128, 256, 512, and 512, stride sizes of 2, and padding set to 1 for all layers. The decoder also contains four transposed convolutional layers with batch sizes of 512, 256, 128, and 1, stride sizes of 2, and padding set to 1. Gabor filters were used in the first convolution layer of our model (Luan et al., 2018) (Figure 2), Research has demonstrated that filters are often redundantly learned in CNN, and the most fundamental filter can be replaced by the Gabor filter. Considering the directivity of raster images and the training complexity of CNNs, the frequency and orientation of the Gabor filter used in this study are similar to those found in the primary visual cortex of mammalian vision systems (Nandy and Banerjee, 2012).

**Figure 3:**
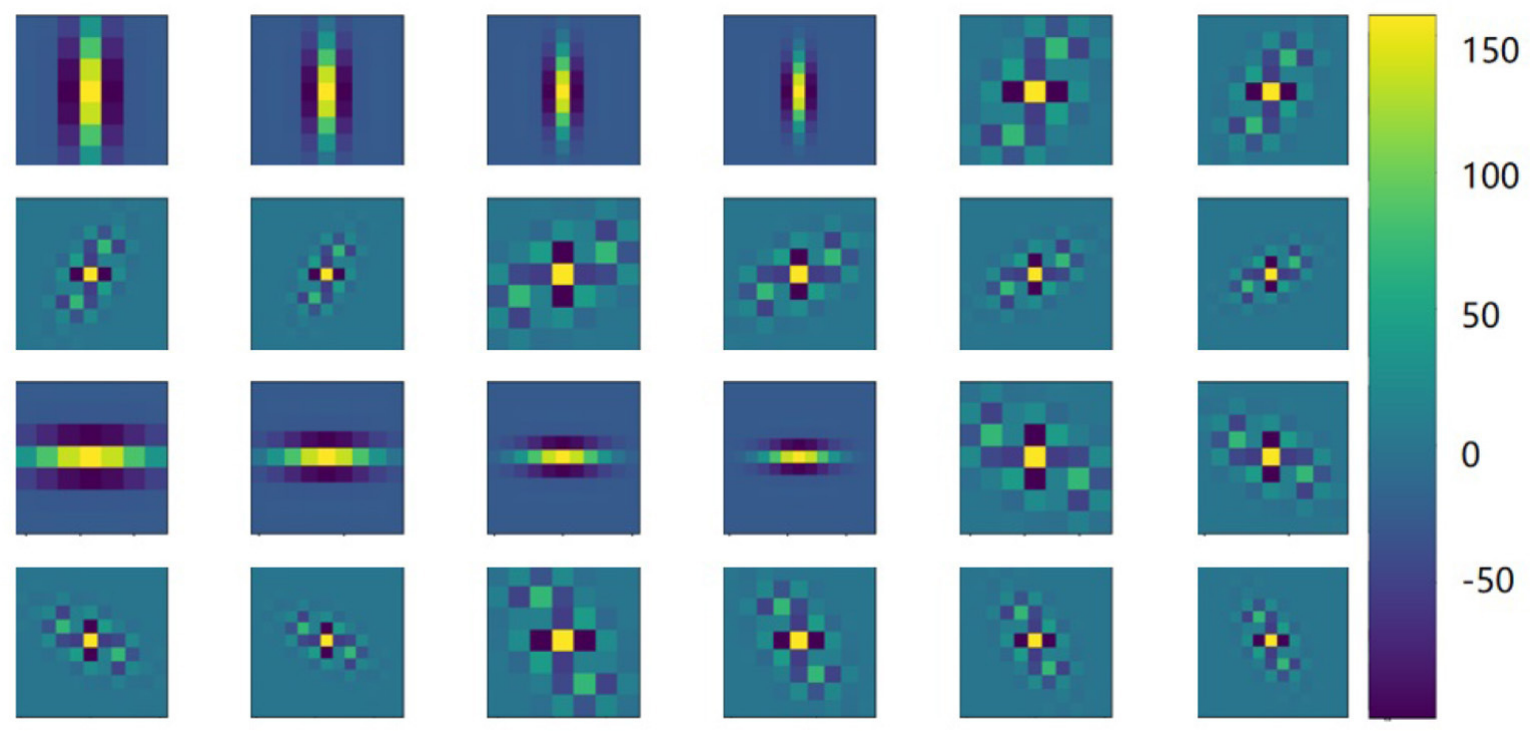
Features with Gabor filters. Illustrations of latent features using Gabor filter with rotated (row, 30-degree step) and scaled (columns, 7, 9, 11, and 13) images. The color indicates the magnitude of the Gabor filter coefficients.

**Figure 4:**
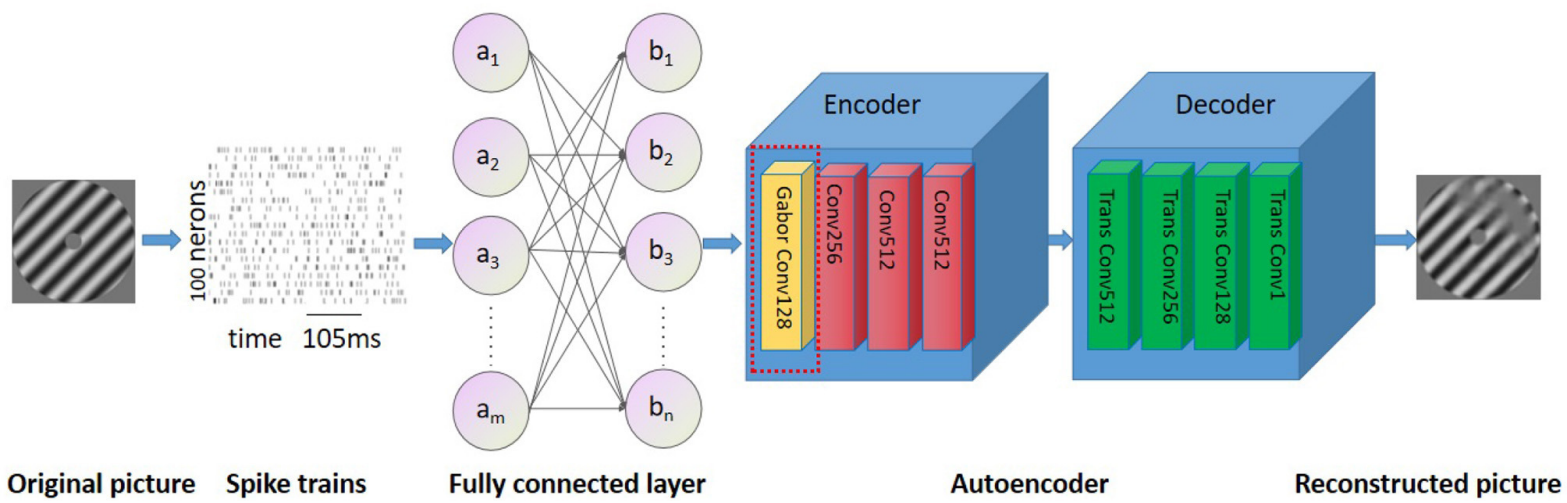
Overview of the neural network. The proposed network consists of two fully connected layers followed by a Gabor autoencoder.

Gabor convolution neural network is a deep neural network using Gabor orientation filters (GoFs), which can produce feature maps to enhance directions and scales information (Figure 3). In addition, GoFs are generally used to model receptive fields of simple cells of the visual cortex. This way, the deep learning model can be strengthened while learning fewer parameters. Raster images with directions and scales could be better fitted when convolutional neural networks are applied (Figure 4).

### 2.4. Loss function

We designed a new loss function and set receptive field properties of the weight matrix, which can be adjusted according to the position of the receptive field. Applying the weight matrix of the receptive field to the loss function can make the different definitions in different parts of the reconstructed images and give higher weight to the area of attention of the receptive field. Due to the need to measure the structural similarity between the original image and the reconstructed image, we fused the Structure Similarity Index Measure (SSIM) in the loss function. SSIM is described in detail in this article (Wang et al., 2004). The comparison measurements are luminance, contrast, and structure. They are described as follows:


(1) $$\begin{array}{l} l_{luminace}\left(x,y\right)=\frac{2\mu_{x}\mu_{y}+c_{1}}{\mu_{x}^{2}+\mu_{y}^{2}+c_{1}} \end{array}$$

(2) $$\begin{array}{l} c_{contrast}\left(x,y\right)=\frac{{2\sigma }_{x\;}\sigma_{y}+c_{2}}{\sigma_{x}^{2}+\sigma_{y}^{2}+c_{2}} \end{array}$$

(3) $$\begin{array}{l} s_{structure}\left(x,y\right)=\frac{\sigma_{xy}+c_{3}}{\sigma_{x}\sigma_{y}+c_{3}} \end{array}$$

(4) $$\begin{array}{l} SSIM\left(x,y\right)={\left[l\left(x,y\right)\right]}^{\alpha }{\left[c\left(x,y\right)\right]}^{\beta }{\left[s\left(x,y\right)\right]}^{\gamma } \end{array}$$

where x represents the reconstructed image and y represents the target image. μ_x_ is the mean of x, σ_x_ is the standard deviation of x, and σ_xy_ is the covariance between x and y. α, β, and γ represent the weights of brightness, contrast, and structural similarity in image reconstruction, respectively. Generally, these weights are equally important, so they are set to 1 and *c*_3_ = *c*_2_/2. The SSIM and MSE can be described as follows:


(5) $$\begin{array}{l} SSIM\left(x,y\right)=\frac{\left(2\mu_{x}\mu_{y}+c_{1}\right)\left({2\sigma }_{xy}+c_{2}\right)}{\left(\mu_{x}^{2}+\mu_{y}^{2}+c_{1}\right)\left(\sigma_{x}^{2}+\sigma_{y}^{2}+c_{2}\right)} \end{array}$$

(6) $$\begin{array}{l} MSE=\frac{1}{I_{h}\times I_{w}}\overset{H}{\underset{i=1}{\sum }}\overset{W}{\underset{j=1}{\sum }}{\left(X_{1}\left(i,j\right)-X_{2}\left(i,j\right)\right)}^{2} \end{array}$$

The boundedness of SSIM is [0,1], the higher the value is, the more similar the two images are. In order to minimize the loss function, we defined SSIM loss as LSSIM = -SSIM(x,y). Our loss function is designed as follows:


(7) $$\begin{array}{l} L={\mu L}_{SSIM}+\left(1-\mu \right)WL_{MSE} \end{array}$$

**Figure 5:**
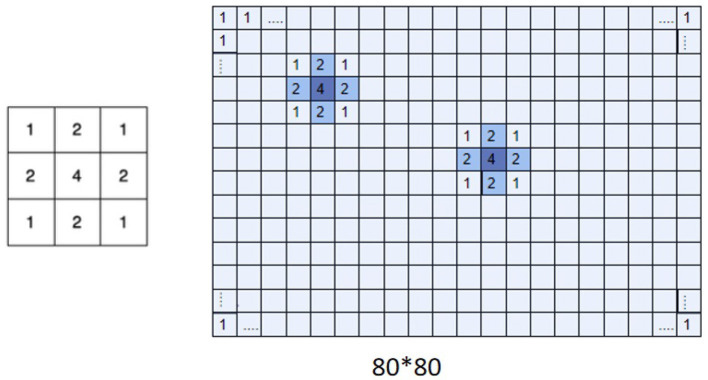
Gaussian kernel and receptive field weight matrix. Multiplication of W matrix **(right)** with Gaussian kernel **(left)**. The W matrix represents the importance of the different regions of an image. The figure displays a weight matrix of size 80×80.

W is a matrix with receptive field properties in Equation (7) and the picture's size determines the W matrix's size (Figure 5). We used a Gaussian kernel as the spatial weight matrix. The Gaussian kernel positions are obtained from the receptive field positions, which are already included in the dataset. In the data of this experiment, there are no cases of two receptive field positions being completely duplicated. Still, in implementation, the weights of overlapping receptive field positions are added up to indicate that the reconstruction weight is greater where there are more receptive fields. The parameter μ in the equation is a hyperparameter regarding the weights, which can be adjusted to configure the proportion of SSIM and weighted MSE to achieve better reconstruction results. The data shown in the table were obtained with μ = 0.1. When information on the spike trains is insufficient to reconstruct a high-resolution image, the weighted loss function can reconstruct images discriminately, where the receptive field region has a higher weight.

### 2.5. Model performance evaluation

The performance of our method was compared with the CNN auto-encoder with the mean square error (MSE), peak signal-to-noise ratio (PSNR), visual information fidelity, pixel domain version (VIFP) (Han et al., 2013), and SSIM loss function. MSE describes the absolute difference of every pixel, the PSNR describes the global quality, and the PSNR is defined as


(8) $$\begin{array}{l} PSNR=10\cdot {log}_{10}\left(\frac{P^{2}}{MSE}\right) \end{array}$$

where P presents the maximum pixel value (255 for 8-bit images). The VIFP quantify the information shared between the test and the reference images, and the SSIM captures the structure similarity, for evaluating the reconstruction results. It is worth noting that among the four metrics, MSE indicates better reconstruction performance with smaller values, while SSIM, PSNR, and VIFP indicate better performance with larger values.

#### 2.5.1. Code and datasets available

The code and datasets are available. The code is available at https://github.com/WYCAS/S2INet. The stimulation and response of macaque V1 and Salamander retina can be found here, respectively: http://crcns.org/data-sets/vc/pvc-8, https://datadryad.org/stash/dataset/doi:10.5061/dryad.4ch10.

## 3. Results

**Figure 6:**
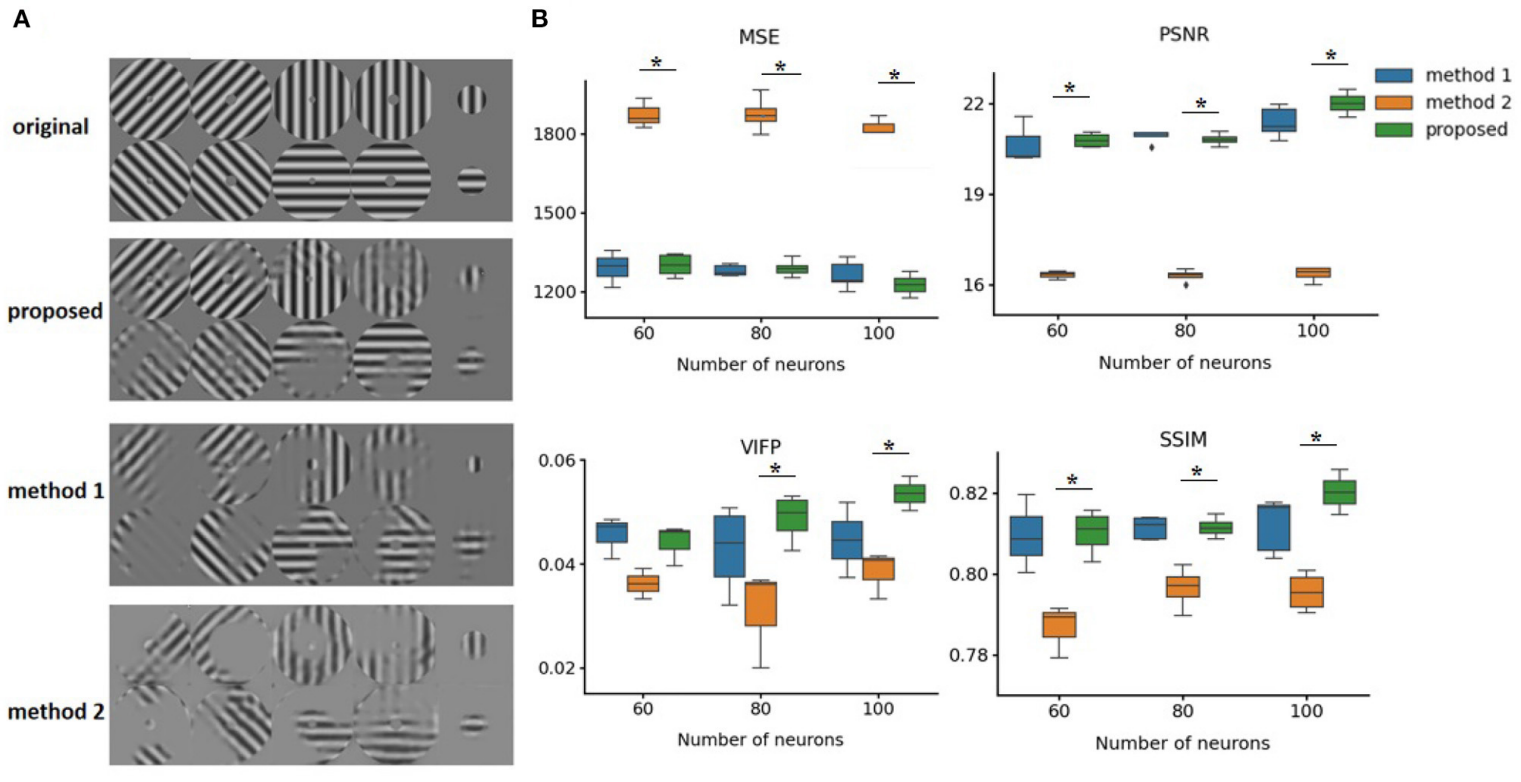
Comparing performance of different reconstruction methods. **(A)** Original neutral images and reconstructed images. Method 1 and Method 2 are both based on autoencoder structures, but use MSE and SSIM loss functions, respectively. **(B)** Reconstruction loss for original images using different methods. *p < 0.05 compared proposed method and method 2 in all four indicators when using 100 neurons. MSE, Mean-Squared Loss; SSIM, Structure Similarity Index Measure; PSNR, Peak signal-to-noise ratio; VIFP, visual information fidelity and pixel domain. Error bars represent 95% confidence interval.

The performance of our method was evaluated on two open source datasets, including macaque primary visual cortex and salamander retina spike trains. We did not use a simulator and chose real datasets to train our model. We evaluated our method on images of gratings that contain four orientations and different degrees in diameter. Figure 6 shows the reconstruction effects of our approach. Our model method outperforms the other two methods. Due to the location of the receptive field, there were other reconstruction effects in the different regions. The first method (method 1) was based on a CNN autoencoder with an MSE loss function, and the second method (method 2) was based on the SSIM loss function (Zhang et al., 2020).

**Figure 7:**
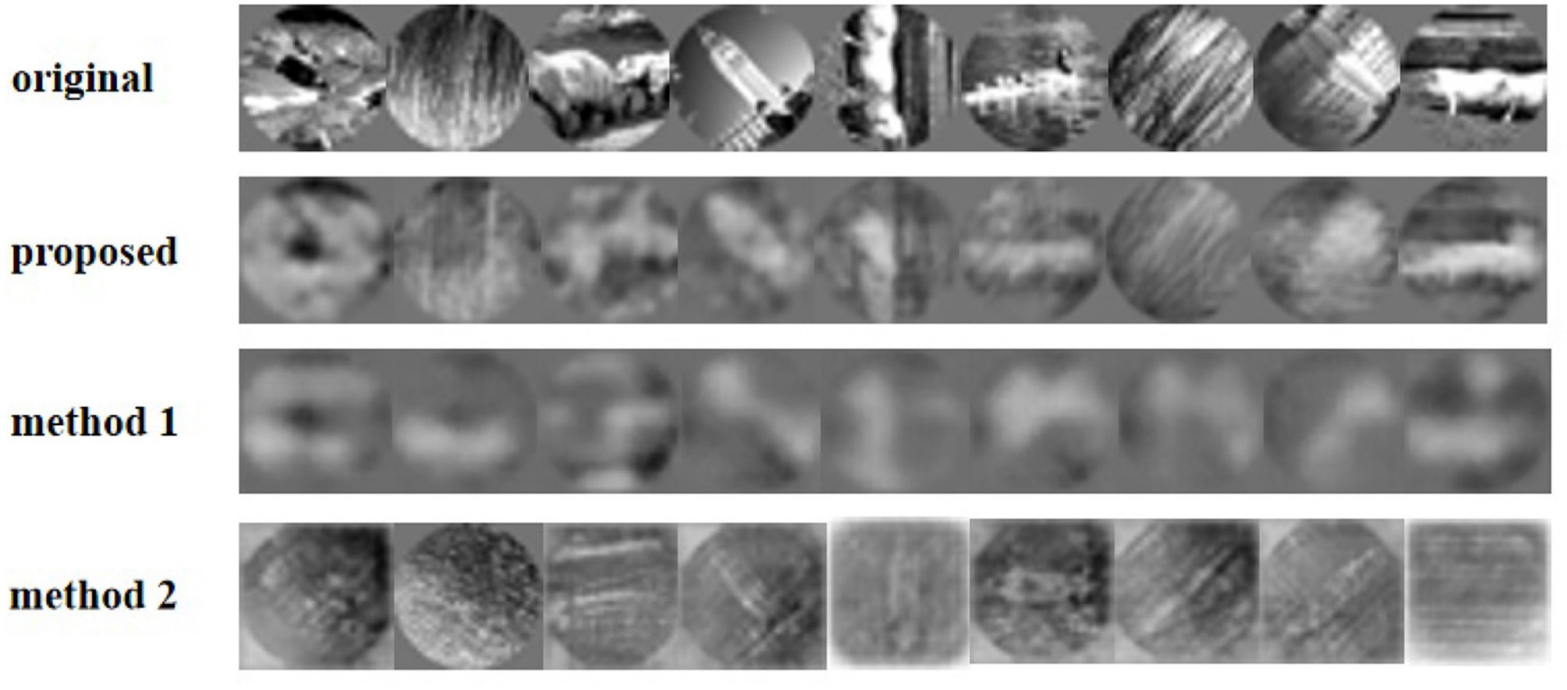
Reconstruction of the natural images using different methods from V1 neurons.

Furthermore, we tried to reconstruct natural images from the spike trains (Figure 7). Affected by the amount of reconstruction information, the resolution of the reconstructed image is reduced to 32*32 pixels grayscale. Figure 7 shows the reconstructed images from macaque V1 spike trains compared to other methods. Due to the influence of input data information and the complex structure of reconstructed images, the reconstructed images are not clear in detail. However, our model method outperforms the other two methods. Especially, method 2 with SSIM loss function has difficulty in reconstructing images. For comparison with macaque V1 data, Table 1 shows the average of 756 images by four typical criteria of reconstructed images. The MSE in the table is obtained by summing the MSE values of all the pixels.

**Table 1:** Performance of our method compared with other methods from macaque V1 spike trains.

**Method**	**MSE**	**PSNR**	**VIFP**	**SSIM**
Method 1	3635.02	13.0551	0.0538	0.5803
Method 2	10174.72	8.7412	0.0439	0.5359
**Proposed**	**3479.79**	**13.1003**	**0.0566**	**0.5945**

The bold values indicates the best-performing value among the three methods.

**Figure 8:**
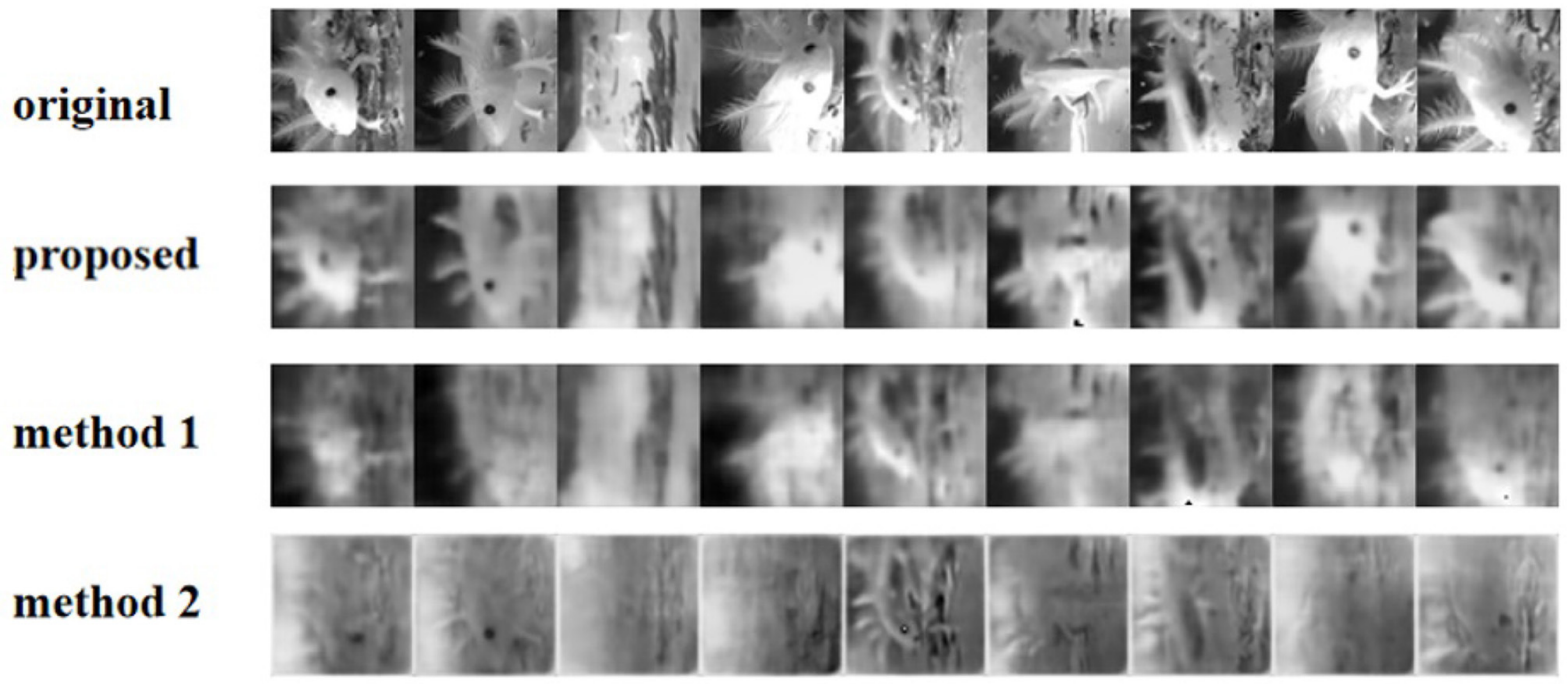
Reconstruction of the video frames using different methods from V1 neurons.

To further test the generalization capability of our method, we performed experiments on responses based on RGCs data (Figure 8). According to the result, our approach has a better reconstruction effect in detail.

Table 2 shows the performance of our method compared with the other two methods based on the salamander RGCs data. Compared to the method described in the study, our method uses Gabor convolution instead of ordinary convolution. It employs a loss function with receptive field weights, and our model suggests that Gabor autoencoder architecture with a weighted loss function enables precise reconstruction. The proposed method can do well in presenting the reconstruction details, especially in complex stripe features of animals or scenes. However, its performance is still poor for stimuli from complex visual images, This may be due to each natural image's short incentives and complex features. The results show the weighted loss with properties of the receptive field for our deep image reconstruction model to achieve perceptually similar reconstructions.

**Table 2:** Reconstructed video frames from RGCs spikes with other methods.

**Method**	**MSE**	**PSNR**	**VIFP**	**SSIM**
Method 1	7623.06	9.3023	0.0554	0.7281
Method 2	2234.36	14.6389	0.2183	0.8088
**Proposed**	**2108.21**	**14.8911**	**0.2316**	**0.8174**

The bold values indicates the best-performing value among the three methods.

## 4. Conclusion

Our approach could remarkably decode visual content from spike trains of RGC and V1 neurons combining the receptive field into a neural network. The brain-inspired model consists of a fully connected layer and a Gabor autoencoder. A loss function with a receptive field weighted matrix was combined with the Gabor auto-encoder, which is critical for our model. As far as we know, this is the first time that receptive field properties were combined into a loss function. The results demonstrated that the brain-inspired method outperforms current other models.

## 5. Discussion

We proposed an innovative brain-inspired model to reconstruct the static image and dynamic video content from neural spike trains of RGC and V1. The model is end-to-end, extracting information from the spike train, and reconstructing images. Our model integrated a loss function with a receptive field weighted matrix inspired by neural computation in a visual system into a Gabor auto-encoder. Our model outperforms other neural networks, demonstrating the great potential of a brain-inspired model to solve the challenge in AI. We further notice that the effect of image reconstruction from macaque V1 and salamander RGC spike trains is different, which is probably caused by hierarchical processing from RGC to V1 neurons, and the sample size of neural spike trains. Future research should strive to improve the model by integrating continuous visual neural signals, which contain logical continuity of visual information.

The development of deep learning has achieved great success in various complex tasks, from natural image classification to natural language processing, and brought AI to the spotlight of broad research communities and commercial users (Christensen et al., 2022). The brain-inspired model, including the auto-encoder and the weighted loss function, demonstrated remarkable promise for next-generation AI with biological interpretability. First, brain-inspired decoders can aid researchers in gaining a deeper understanding of the neural mechanisms involved in perception and decision-making. By modeling the neural processes that underlie these cognitive functions, brain-inspired decoders can assist researchers in developing more precise and comprehensive models of the brain. Second, these decoders can facilitate the development of advanced computer vision systems and enhance the performance of artificial intelligence (AI) systems. By modeling the neural processes associated with visual perception, these decoders can improve machines' abilities to recognize and interpret visual information accurately and assist AI systems in more effectively adapting to complex environments. The brain-inspired model is also probably a key technology for implementing artificial systems that solve problems that the brain solves, like robotics control, self-driving, smell-sensing, dynamic vision sensors, and bio-hybrid systems for brain repair.

## Data availability statement

The raw data supporting the conclusions of this article will be made available by the authors, without undue reservation.

## Ethics statement

The animal study was reviewed and approved by the Albert Einstein College of Medicine at Yeshiva University.

## Author contributions

WL and XL contributed to conception and design of the study. RH organized the dataset. WL performed the statistical analysis and code and wrote the first draft of the manuscript. CD, SZ, YL, CH, and WC wrote sections of the manuscript. All authors contributed to manuscript revision, read, and approved the submitted version.
