# Supplementary material for: The brain-inspired decoder for natural visual image reconstruction
Source: Front Neurosci. 2023 May 2;17:1130606. doi: 10.3389/fnins.2023.1130606 (PMC10185745; doi:10.3389/fnins.2023.1130606)
Supplement: Supplementary file 1 [file Data_Sheet_1.PDF]

CRCNS.org pvc-8 (primary visual cortex, number 8) data description
Version 0.8, Nov 23, 2015

This data consist of multi-electrode recordings from V1 in anesthetized macaque monkeys, while natural images and gratings were flashed on the screen. Recordings were performed using the "Utah" electrode array. Natural images were presented at two sizes, 3-6.7 degrees and windowed to 1 degree, to quantify surround modulation. Experimental procedures and stimuli are fully described in the associated paper:

> Ruben Coen-Cagli, Adam Kohn, Odelia Schwartz; "Flexible Gating of Contextual Influences in Natural Vision." Nature Neuroscience, 2015. doi:10.1038/nn.4128

Format of the data

Data from ten recording sessions are included. They are stored in mat files, with names 01.mat ... 10.mat. The data were recorded from three animals (session 01 from animal 1; 02-07 from animal 2; session 08-10 from animal 3). Total data size is about 300MB. The data from each session includes the following Matlab variables:

- resp_train: array of spiketrains from all neurons to all images/trials [#neurons #image #trials #milliseconds]. Time 0 is stimulus onset. Stimuli are presented for 106 ms; the times are for when the stimulus is on the screen.

- resp_train_blk: array of spiketrains during the subsequent blanks, from all neurons to all images/trials [#neurons #image #trials #milliseconds]

- INDCENT: array of 1s and 0s, where 1s denote the centered (and spike-sorted) neurons [#neurons]

- RF_SPATIAL: for each neuron, the parameters of the 2D Gaussian fit to the spatial RF (x position, y position, var(x), var(y), fit quality R^2) [#neurons 5]; position is relative to the image center.

- RF_TUNING: for each neuron, the parameters of the steerable pyramid used to build the filters (scale, orientation bandwidth, transition width, preferred orientation) [#neurons 4]

- P_HOMOG: for each neuron and image, the probability that the image is homogeneous [#neurons #images]

- images: a cell array of images (the first 540 are natural, alternating one small one big; the remaining images are gratings). Further details about the images and gratings parameters are provided in the associated Matlab code described below.

<u>Notes:</u>

- The images were centered inside the aggregate receptive field of the array, therefore images were not well centered for some individual channels (typically 50-75%); the RF position relative to image center is included with the data files, as explained above. Spike sorting was performed only on well-centered channels.

- Preprocessing for the original publication included shifting the spike trains by 50 ms, to account for response latency. This has not been performed on the data provided here, but is illustrated in the associated Matlab script.

- RF_SPATIAL is not available (set to NaN) for files 08 to 10

- RF_TUNING and P_HOMOG are not available (set to NaN) for non-centered neurons

- P_HOMOG not available (set to NaN) for gratings images.

- Pyramid (we use the function "buildSCFpyramid.m" from the Steerable Pyramid Toolbox, available on Eero Simoncelli's website): the parameter "orientation bandwidth" takes values 1 (pyramid with 4 orientations), 2 (with 8 orientations), 3 (with 16 orientations). The parameter "transition width" takes values {1,2,3,4} corresponding to {0.5,1,2,3} as the last input to buildSCFpyramid.

How to get started

All data files can be load directly into Matlab. Examples of how to load the data and interpret the variables are provided in the associated Matlab script:

main_LoadAndParse.m

This code loads one example dataset (spike trains, stimuli, and additional variables described above). It applies some pre-processing, and creates separate variables to store the spike count responses to different image sets. It then shows how to compute surround suppression indexes and how to classify images as homogeneous or heterogeneous. Furthermore, it shows how to compute tuning properties from the responses to gratings.

Conditions for using the data

These data are being offered to allow other investigators to explore basic questions about the representation of visual information in V1 populations during natural image stimulation. Ideally, we are seeking to actively collaborate with other groups on this research goal, which could lead to new discovery and joint publications. However, we are placing no restrictions on the use of the present data, except: (1) Please consult Adam Kohn (adam.kohn@einstein.yu.edu) before publication; (2) If you wish to publish any results using these data, please include the following statement in either the Methods or Acknowledgement section:

"The data were collected in the Laboratory of Adam Kohn at the Albert Einstein College of Medicine and downloaded from the CRCNS web site."

Also, any publications made using the data should cite the Coen-Cagli et al., 2015 paper mentioned above, and also cite data set using the following:

> Adam Kohn, Ruben Coen-Cagli (2015). Multi-electrode recordings of anesthetized macaque V1 responses to static natural images and gratings. CRCNS.org. http://dx.doi.org/10.6080/K0SB43P8

Getting help

Questions that could be of general interest should be posted to the CRCNS.org forum. Also, comments/suggestions/bugs can be sent to: Adam Kohn; adam.kohn@einstein.yu.edu
